# Construction of a 10-gene prognostic score model of predicting recurrence for laryngeal cancer

**DOI:** 10.1186/s40001-022-00829-2

**Published:** 2022-11-14

**Authors:** Yanan Liu, Zhiguang Gao, Cheng Peng, Xingli Jiang

**Affiliations:** 1 Department of Gastroenterology, Harbin Institute of Technology Heilongjiang Hospital, 82 Zhongshan Road, Harbin, 150036 Heilongjiang People’s Republic of China; 2 Department of Otorhinolaryngology, Heilongjiang Provincial Hospital Affiliated to Harbin Institute of Technology, 82 Zhongshan Road, Harbin, 150036 Heilongjiang People’s Republic of China

**Keywords:** Laryngeal cancer, Recurrence, Prognosis

## Abstract

We constructed a prognostic score (PS) model to predict the recurrence risk in patients previously diagnosed with laryngeal cancer (LC). Here the training dataset, consisting of 82 LC samples, was downloaded from The Cancer Genome Atlas (TCGA). The PS model then divided the LC samples into high- and low-risk groups, which predicted well the survival time of LC in three datasets (TCGA dataset: AUC = 0.899; GSE27020: AUC = 0.719; and GSE25727: AUC = 0.662). Therefore, the PS model based on the 10 genes and its nomogram is proposed to help predict the recurrence risk in patients with LC.

**Supplementary Information:**

The online version contains supplementary material available at 10.1186/s40001-022-00829-2.

## Introduction

Laryngeal cancer (LC) has been identified as the one of the most common types of head and neck cancers, which resulted in approximately 11,150 new cases in the United States in 2018 [1]. During the past decades, various treatment strategies have been devised for treating LC. However, the 5-year overall survival (OS) of patients with LC remains unsatisfactory [2]. According to the SEER database from 2006 to 2012, the 5-year OS of LC remained as low as 60.7%, which has not increased significantly in the last few decades [3]. Furthermore, the local recurrence of LC is common among patients, such as those with moderately or poorly differentiated squamous cell carcinoma, in addition to the thyroid cartilage plate invasion. Hence, comprehensive treatment and closer follow-up should be given to these patients [4]. Nevertheless, the identification of novel prognostic gene markers that can help distinguish the recurrence risk in patients with LC is vital for improving the OS of patients with LC.

In recent decades, the occurrence of next-sequencing technologies has made rapid disease and recurrence detection possible. Notably, existing evidence has indicated that many gene biomarkers have predictive values for LC. Likewise, Zhang et al. [5] indicated that five genes (*EMP1*, *HOXB9*, *DPY19L2P1*, *MMP1*, and *KLHDC7B*) had the potential function to predict LC recurrence. Cury et al. [6] also argued that *DSG2* overexpression was associated with shorter OS. And, it is also indicated that high plasma protein levels of *DSG2* indicated its detection in liquid biopsy, which is proposed to be applied as a recurring biomarker for LC. Pedro et al. [7] have also reported that *ALCAM* overexpression was an independent biomarker for predicting recurrence of laryngeal squamous cell carcinoma in patients. Nevertheless, although previous studies have identified numerous gene targets that account for the LC recurrence, further investigations are needed to explore the effect of these featured genes on the recurrence risk in patients with LC.

Therefore, according to the multiple bioinformatics data, we screened the genes significantly correlated with recurring LC using meta-analysis and L1-penalized optimization algorithm. Then, we constructed the risk model for predicting recurrence risk in patients with LC.

## Method

### Data source

The mRNA sequencing data of head and neck samples (including 604 samples) were obtained from The Cancer Genome Atlas (TCGA) database (https://tcga-data.nci.nih.gov/docs/publications/tcga/?) based on the Illumina HiSeq 2000 RNA Sequencing platform. The positions of the 604 samples were in the alveolar ridges (*n* = 18), tongue roots (*n* = 30), buccal mucosa (*n* = 22), floor of the mouth (*n* = 67), hard palates (*n* = 8), hypopharynx (*n* = 9), larynx (*n* = 138), lips (*n* = 3), mouth (*n* = 38), tongue (*n *= 158), oropharynx (*n* = 10), and tonsils (*n* = 45). The rest of the samples were from uncertain tumor locations. Among the 138 throat samples, we screened 82 LC samples with recurrence and prognosis information (28 and 54 samples with and without recurrence, respectively) in our study.

Additionally, we searched for validation dataset using the keyword “larynx cancer” from the National Center for Biotechnology Information Gene Expression Omnibus database (http://www.ncbi.nlm.nih.gov/geo/). The screening standards were as follows: (1) gene expression profile data, (2) the samples were from the tumor tissue specimen of patients, (3) human expression profile data, and (4) the samples with information of recurrent or non-recurrent prognosis. Two validation datasets were obtained. One was GSE27020 that composed of 109 LC tissue samples (34 and 75 samples with and without recurrence, respectively) based on the Affymetrix Human Genome U133A, the Array platform, and the other one was GSE25727 that included 56 LC tissue samples (17 and 39 samples with and without recurrence, respectively) based on the Illumina HumanRef-8 WG-DASLv3.0 platform.

### Screening of differentially expressed genes

A meta-analysis on TCGA dataset, GSE27020 and GSE25727, was conducted using an ES function of MetaDE [8] (version: 1.0.5, https://cran.r-project.org/web/packages/MetaDE) in R3.4.1 to screen the differentially expressed genes (DEGs) [9]. Subsequently, we screened for DEGs [9] that showed consistent expression in these two datasets between samples with recurrence and those without recurrence by calculating the tau^2^, Q, and Qpval values (criterion for judgment; tau^2^ = 0 indicates that each research object is homogeneous and unbiased; the statistic Q obeys the Chi-square test with a degree of freedom of k-1, whereas Qpval  > 0.05 indicates that each research object is homogeneous and unbiased). Then, the false discovery rate (FDR) value was obtained using multiple test corrections. FDR < 0.05 showed that the difference was significant. Additionally, each dataset was calculated to express the fold change, after which several parameters were selected, and the threshold value was set. The set parameters were as follows: (1) To ensure that the source of each selected characteristic gene was homogeneous and unbiased (that the expression of each featured gene in each data set was consistent), Tau^2^ = 0 and Qpval  > 0.05 were selected as homogeneity test parameters. (2) FDR < 0.05 was selected as the significant threshold of expression difference between the genomes. (3) After screening with log2 FC, the genes having similar direction of difference (with the same symbol of log2 FC) were retained. After combining multiple screening parameters, we set the selection of threshold parameters:

I. We ensure that the source of each selected characteristic gene is homogeneous and unbiased, that is, the expression in each data set is consistent, so tau^2^ = 0 and Qpval > 0.05 are selected as homogeneity test parameters;

II. FDR < 0.05 was considered as the threshold of significant difference in expression between gene groups;

III. Combined with Log2FC for screening, we retained genes with the same direction of difference (consistent Log2FC symbols) in the three datasets.

The threshold was set to a false discovery rate < 0.05. Then, the Gene Ontology Biology Process (GO-BP) annotation analysis and Kyoto Encyclopedia of Genes and Genomes (KEGG) pathway analysis were conducted for these DEGs with consistency.

### Establishment and verification of a risk assessment model

On the basis of the DEGs, we conducted univariate Cox regression analysis in the survival package [10] (version 2.4, http://bioconductor.org/packages/survivalr/) to screen DEGs significantly related to the prognosis in TCGA dataset. The multivariate Cox regression analysis was then used to screen DEGs that can be used as independent prognostic factors. The log-rank *P* < 0.05 was also regarded as the threshold of significant correlation.

Furthermore, the Cox proportional hazard model [11] based on the L1-penalized (Lasso) in the penalized package (version 0.950; http://bioconductor.org/packages/penalized/) [12] of the R3.4.1 language was used to screen out the optimized prognostic-associated signature DEG combinations based on the aforementioned DEGs related to the prognosis [13]. Then, on the basis of the prognostic coefficient of prognosis-related DEGs, the prognostic score (PS) prediction model was established in the training dataset using the following formula:

$${\text{ps}} = \sum {\beta {\text{DEGs}}} \times {\text{ExpDEGs,}}$$

where *β*DEGs refer to the prognostic coefficient of the optimized DEGs in the Lasso algorithm and Exp_DEGs_ represent the expression of the corresponding DEGs in the training dataset.

With the median PS as the dividing point, the samples in TCGA training dataset were further categorized into high- and low-risk groups. After that, the Kaplan–Meier (KM) [14] survival curve in the R3.4.1 language survival package (version 2.41–1) [10] was used to measure the association between the risk model and prognosis. Simultaneously, we screened these optimized DEGs from the validation dataset (GSE25727 and GSE27020). Then, the PS score of each sample was obtained using the PS calculation method. The validation dataset samples were also separated into high- and low-risk sample groups in the same manner as in TCGA dataset samples. Thereafter, the KM curve method of the survival package (version 2.41–1) [10] in the R3.4.1 language was used to evaluate the relationship between the high- and low-risk groups, compared with the actual survival prognosis information from the validation dataset samples.

### Screening of independent prognostic clinical factors for performance evaluation

Combining the clinical factors including recurrence, age, gender, pathologic (M, N, and T), pathologic stage, grade, alcohol history, angiolymphatic invasion, and perineural invasion in TCGA (Additional file 1: Table S1), we used univariate and multivariate Cox regression analysis methods in the R3.4.1 language survival package (version 2.41–1) [10] to screen the independent prognostic clinical factors. The threshold was set to log-rank *P* < 0.05. Next, to further explore the correlation between independent factors and prognosis, the nomogram with 3- and 5-year survival rates was constructed using the RMS software package (version 5.1.2; https://cran.r-project.org/web/packages/rms/index.html) in R3.4.1 [9, 15].

Next, the PS and risk models were compared using the area under the receiver-operating characteristic curve (AUROC) [14] and the concordance index (C-index). Additionally, the AUROC is a quantitative indicator of the receiver-operating characteristic (ROC) curve, which was calculated using the pROC in the R3.4.1 language (version 1.14.0, https://cran.r-project.org/web/packages/pROC/index.html). In contrast, the C-index is referred to as the scores of all individual pairs correctly sorted on the basis of the Harrell C statistics [16] to predict the survival time [17]. It was calculated using the survcomp package (http://www.bioconductor.org/packages/release/bioc/html/survcomp.html) in the R3.4.1 language.

## Results

### Identification of DEGs

**Fig. 1:**
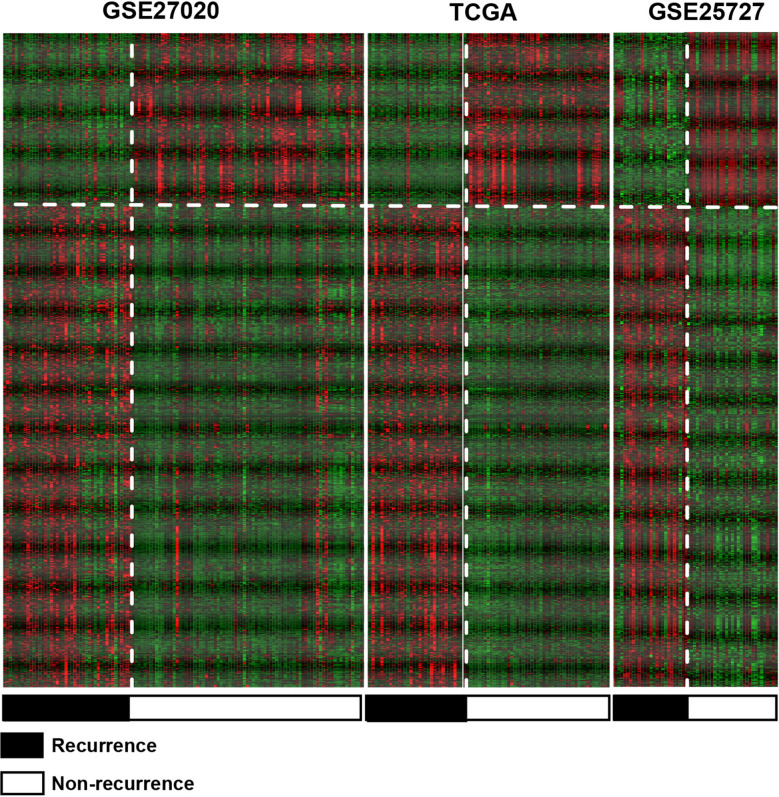
A two-way hierarchical clustering heat map of TCGA, GSE27020 and GSE25727, datasets based on consistent DEGs

A total of 981 DEGs were detected among TCGA datasets, GSE25727 and GSE27020, which contained 347 down-regulated genes and 634 up-regulated genes (Fig. 1 and Additional file 2: Table S2). The DEGs were significantly different among the various types of samples from the three datasets. This result indicated that the DEGs expressed significant difference among the three datasets.

**Fig. 2:**
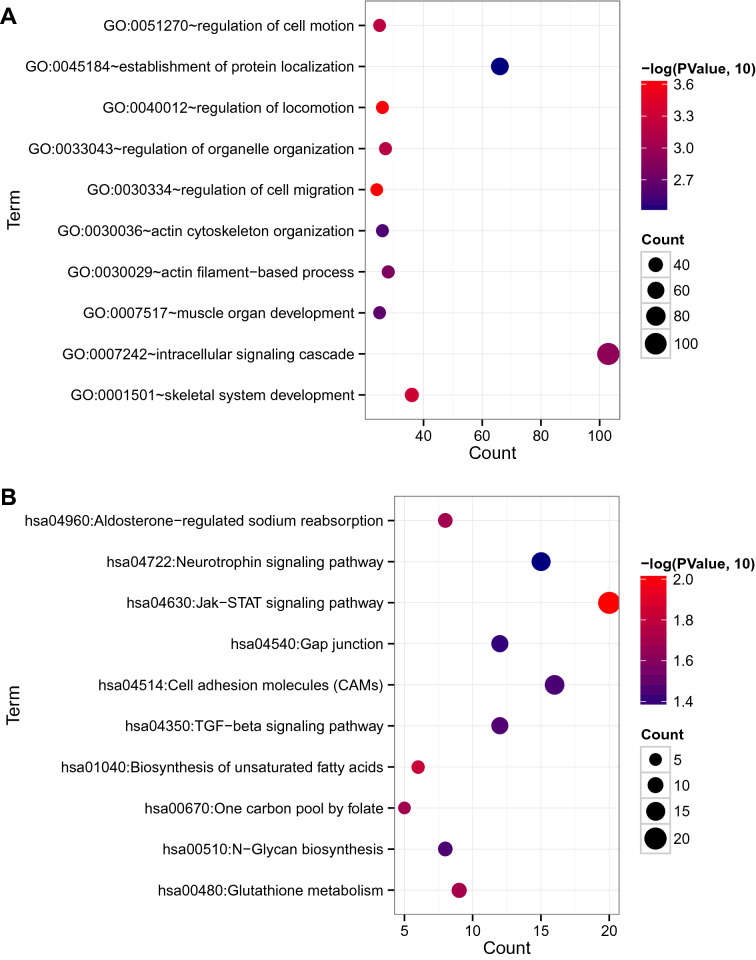
GO-BPs (**A**) and KEGG pathways (**B**) involved in DEGs

The GO results suggested that these genes are involved in 41 GO-BP terms, such as regulation of cell migration (*P* = 2.26E−04) and regulation of locomotion (*P* = 2.48E−04). Simultaneously, these DEGs were enriched in 10 KEGG pathways, including the Jak–STAT signaling pathway (*P* = 9.44E−03) (Fig. 2 and Table 1).

**Table 1:** GO biological process and KEGG signal pathway significantly related to target genes

Category	Term	Count	*P* value	Genes
Biology Process	GO:0030334 ~ regulation of cell migration	24	2.26E−04	DLC1, PARD6B, IRS2, FLT1
	GO:0040012 ~ regulation of locomotion	26	2.48E−04	DLC1, PDGFB, ENPP2, TAC1,
	GO:0001501 ~ skeletal system development	36	4.90E−04	TUFT1, GNA11, HEXA, HOXD12
	GO:0051270 ~ regulation of cell motion	25	6.45E−04	SORT1, PDGFRB, PBX1, IGFBP3
	GO:0033043 ~ regulation of organelle organization	27	6.92E−04	DLC1, SHROOM2, CAPZA1, TAC1
	GO:0007242 ~ intracellular signaling cascade	103	1.24E−03	RAB9A, ADCY7, PLEKHM1, GNA11
	GO:0030029 ~ actin filament-based process	28	1.53E−03	CHEK1, ARF5, TP53TG5, P2RY1
	GO:0007517 ~ muscle organ development	25	2.22E−03	NMUR1, RHOF, CHUK, RAP2B
	GO:0030036 ~ actin cytoskeleton organization	26	2.66E−03	GDI1, FLT1, MCF2, IGF1,
	GO:0045184 ~ establishment of protein localization	66	3.89E−03	RAB9A, APOBEC1, AP1G1, SLC15A2
KEGG pathway	hsa04630:Jak–STAT signaling pathway	20	9.44E−03	CSF3, PTPN6, CTF1, SOCS1
	hsa01040:Biosynthesis of unsaturated fatty acids	6	1.49E−02	BAAT, ELOVL5, HSD17B12, ELOVL2,
	hsa00480:Glutathione metabolism	9	1.95E−02	GGT5, GSTA4, G6PD, RRM2
	hsa04960:Aldosterone-regulated sodium reabsorption	8	2.03E−02	MAPK1, IRS2, MAPK3, IGF1,
	hsa00670:One carbon pool by folate	5	2.07E−02	MTHFD2, MTHFR, SHMT2, MTR
	hsa04350:TGF-beta signaling pathway	12	3.51E−02	INHBB, MAPK1, SP1, ROCK2
	hsa00510:N-Glycan biosynthesis	8	3.60E−02	MAN2A1, B4GALT3, GANAB, MAN1B1
	hsa04514:Cell adhesion molecules (CAMs)	16	3.65E−02	CLDN8, CLDN7, CLDN17, MPZL1,
	hsa04540:Gap junction	12	4.05E−02	MAPK1, PLCB4, GNAI3, ADCY7
	hsa04722:Neurotrophin signaling pathway	15	4.44E−02	PDK1, IRS2, CAMK2G, IRS1

*GO* Gene Ontology, *KEGG* Kyoto Encyclopedia of Genes and Genomes

### Constructing the prognosis prediction model

A total of 206 prognosis-related DEGs were screened using univariate Cox regression analysis with a threshold of *P* < 0.05 (Additional file 3: Table S3). On the basis of the aforementioned DEGs, we obtained 96 DEGs via the multivariate Cox regression analysis. Subsequently, 10 optimized DEGs (*CD38*, *ZNF212*, *POR*, *CC2D1A*, *GRAMD4*, *FH*, *SLC24A3*, *GATA2*, *FOXD1*, and *MMP10*) were selected using the L1-penalized algorithm (Table 2).

**Table 2:** Information of optimizing DEGs combination

Symbol	Multi-variate Cox regression analysis			LASSO coef
	HR	95%CI	P value	
CD38	0.4053	0.217–0.758	0.0046	− 0.3307
ZNF212	0.0902	0.022–0.370	8.38E−04	− 0.3008
POR	0.0896	0.024–0.331	3.0E−04	− 0.3017
CC2D1A	0.1025	0.020–0.527	6.38E−03	− 0.2154
GRAMD4	0.0667	0.018–0.248	5.27E−05	− 0.3175
FH	0.7175	9.1E−03–0.565	0.0123	− 0.0689
SLC24A3	2.2113	1.160–4.215	0.0159	0.0091
GATA2	5.7842	1.536–21.789	9.49E−0−3	0.0538
FOXD1	3.0618	1.283–7.308	0.0117	0.2088
MMP10	2.0820	1.415–3.064	2.0E−04	0.0044

*DEGs *differentially expressed genes, *HR* hazard ratio, CI confidence

### Evaluation and comparison of the prognostic risk prediction model’s effectiveness

**Fig. 3:**
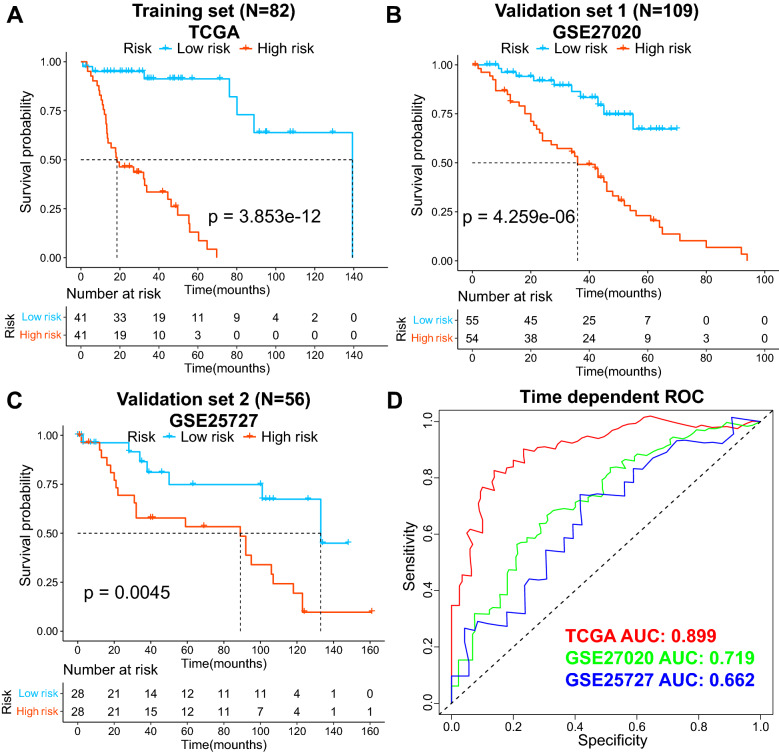
Construction of prognostic risk prediction models. The samples of the **A** training set, **B** validation dataset 1 (GSE27020), and **C** validation dataset 2 (GSE25727) are all based on the KM curve of the PS prediction models and prognosis, with blue and red curves representing low- and high-risk samples, respectively (**D**). The ROC curve of the prediction results was based on the PS prognosis model

As shown in Fig. 3, the *P*S value based on the 10 optimized DEGs could distinctly divide 82 patients with LC into high- and low-risk groups in TCGA training dataset, which indicated that the patients in the high-risk group were related to shorter OS in TCGA dataset (*P* = 3.853e−12). Meanwhile, we obtained the similar results from the validation datasets, which included GSE27020 (*P* = 4.259e−06) and GSE25727 (*P* = 0.0045).

Furthermore, the ROC curves based on the PS prediction model indicated that this PS model accurately predicted the patient survival time in both TCGA dataset (AUC = 0.899) and validation dataset (GSE27020: AUC = 0.719; GSE25727: AUC = 0.662).

### Screening of independent prognostic clinical factors

**Fig. 4:**
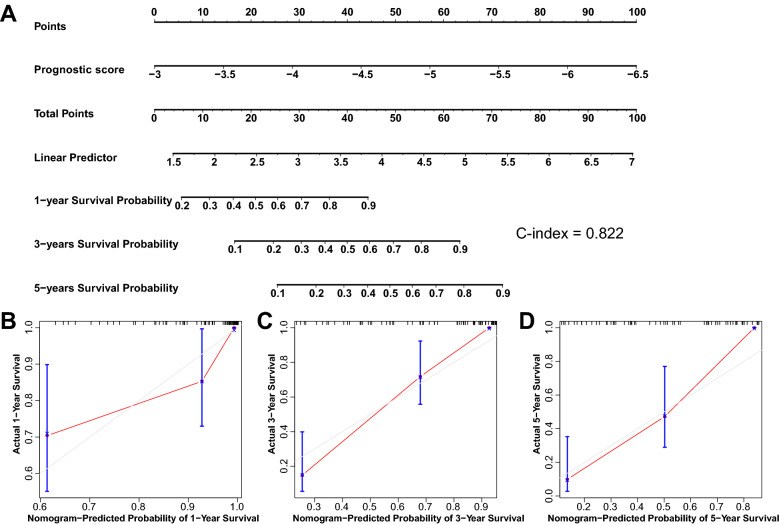
Construction of nomogram to predict the prognostic ability for patients with LC. **A** A nomogram was constructed using the PS model to predict the prognosis for patients with LC. The calibration plots for 1-year (**B**), 3-year (**C**), and 5-year (**D**) survival time

As expressed in Table 3, the PS model was substantially correlated with the LC clinical condition, which was an independent prognostic parameter. Subsequently, the PS model status was included in the nomogram model to predict the 3- and 5-year OS in patients with LC. After that, the score of each index was observed on the point table at the upper apex of the nomogram. Next, the scores of each index were added to estimate the 3- and 5-year survival probability (Fig. 4). These results indicated that the nomogram on the basis of the PS model status had high prediction accuracy for the survival and prognosis of patients with LC.

**Table 3:** Information of clinical factors

Clinical characteristics	TCGA (*N* = 82)	Uni-variables Cox			Multi-variables Cox		
		HR	95%CI	*P*	HR	95%CI	*P*
Age (years,mean ± sd)	60.80 ± 8.29	0.973	0.933–1.015	0.201	0.961	0.875–1.054	0.395
Gender(female/male)	11/71	0.375	0.161–0.872	0.0227	1.008	0.060–16.918	0.996
Pathologic M(M0/M1/–)	78/0/4	–	–	–	–	–	–
Pathologic N(N0/N1/N2/N3/–)	38/17/20/2/5	0.345	0.935–1.935	0.111	1.568	0.753–3.262	0.229
Pathologic T(T1/T2/T3/T4/–)	2/10/24/43/3	0.770	0.511–1.159	0.211	4.321	0.290–64.345	0.288
Pathologic stage (I/II/III/IV/–)	2/5/18/54/3	0.862	0.543–1.367	0.527	0.139	0.006–2.992	0.207
Neoplasm grade(1/2/3)	7/48/26/1	0.923	0.561–1.516	0.75	1.442	0.325–6.407	0.630
Alcohol history(yes/no/–)	57/23/2	0.861	0.432–1.714	0.67	2.703	0.437–16.736	0.285
Angiolymphatic invasion(yes/no/–)	24/35/23	1.184	0.225–2.669	0.684	3.128	0.405–24.172	0.274
Perineural invasion(yes/no/–)	16/41/25	1.037	0.421–2.555	0.938	0.235	0.020–2.818	0.253
PS model status(high/low)	41/41	8.967	4.794–16.77	6.61E-12	86.677	13.996–536.769	1.62E-06
Recurrence (dead/alive)	28/54	1.386	0.740–2.594	0.308	0.807	0.158–4.132	0.797
Recurrence free survival time (months, mean ± sd)	36.91 ± 30.17	–	–	–			

N number, TCGA The Cancer Genome Atlas, *HR* hazard ratio; *CI* confidence

## Discussion

As shown by the previous reports, it is important to detect several crucial gene biology markers associated with the LC survival prognosis, as this could provide a vital theoretical reference for treating patients with LC. Therefore, in our study, a PS model was established on the basis of 10 independent prognostic genes (*CD38*, *ZNF212*, *POR*, *CC2D1A*, *GRAMD4*, *FH*, *SLC24A3*, *GATA2*, *FOXD1*, and *MMP10*). Moreover, the PS model was determined to be an independent recurrence factor for the survival of patients.

Furthermore, LC recurrence seriously affects the survival time of patients with LC. Recently, high-throughput sequencing technologies have also improved the understanding of recurrent gene functions by decoding the genome of patients with LC. Besides, the prediction model of LC recurrence helped in the clinical decision-making. In our study, among the 206 DEGs related to the LC recurrence, 96 independent prognosis-related DEGs were screened using the multivariate Cox regression analysis. We identified 10 metabolic genes associated with prognosis and were further revealed by LASSO-based Cox proportional hazard model analysis to construct the RS survival prediction model, including *CD38*, *ZNF212*, *POR*, *CC2D1A*, *GRAMD4*, *FH*, *SLC24A3*, *GATA2*, *FOXD1*, and *MMP10*. The KM curves showed that the patients with LC in the low-risk group had remarkably better survival than the low-risk group for TCGA dataset (*P* = 3.853e−12). Meanwhile, we observed the similar findings in the validation datasets including GSE27020 (*P* = 4.259e−06) and GSE25727 (*P* = 0.045). A study from Xiang et al. [18] showed a PS model was constructed to predict the recurrence in patients with LC. The PS value demonstrated good accuracy in predicting the relapse with an AUC of 0.859 was at 1 year, 0.822 at 3 years, and 0.815 at 5 years survival predictive accuracy. Besides, Zhang et al. [19] constructed a four-gene signature that could be used to predict the prognosis of patients with LC. It is hypothesized that the four genes might affect the prognosis of patients with LC via mechanisms involved in the immune response and negative regulation of the Wnt signaling pathway. Moreover, Fan et al. [20] indicated that the constructed nomogram of the LC survival risk was good for predicting accuracy, which is helpful for doctors to make a more accurate prognosis evaluation of patients with LC, and can be used to guide and optimize the treatment of patients with LC. Likewise, the 10 independent prognostic genes were used to construct the PS model might be novel biomarkers for risk recurrence of patients with LC. Furthermore, we also constructed a nomogram with C-index of 0.822 using the PS model, which indicated that the nomogram performance has a good concordance with the prediction of 1-, 3-, and 5-year OS. Therefore, the PS model based on the 10 DEGs has the potential ability in the area of prognostic prediction.

In our study, some limitations exist. First, we found that the PS model based on 10 genes had a good predictive ability to predict LC recurrence. However, we failed to determine their detailed mechanisms. Then, only the PS model was screened through the multivariate Cox regression analysis with a threshold of *P* < 0.05. Therefore, we could not analyze other models based on other risk factors. Additionally, our study required large samples and clinical data to confirm whether the model we constructed would accurately distinguish high- and low-risk patients with recurrent LC. Finally, corresponding experimental studies should be conducted to verify the functions of these ten key genes.

## Conclusion

A 10-gene PS model and nomogram are proposed to help predict the recurrence risk in patients with LC.

## Supplementary Information

**Additional file 1.** The clinical factors in TCGA.

**Additional file 2.** The differentially expressed genes among TCGA, GSE25727 and GSE27020 datasets.

**Additional file 3.** Screening of prognosis-related differentially expressed genes using univariate Cox regression analysis.

## Data Availability

The raw data were collected and analyzed by the authors, and are not ready to share their data because the data have not been published.

## Abbreviations

TCGA: The Cancer Genome Atlas
DEGs: Differentially expressed genes
KEGG: Kyoto Encyclopedia of Genes and Genomes
GO-BPs: Gene Ontology Biology Processes
DEGs: Differentially expressed genes
ROC: Receiver-operating characteristic
LC: Laryngeal cancer
PS: Prognostic score

## References

[1] Obid R, Redlich M, Tomeh C (2019). The treatment of laryngeal cancer. Oral Maxillofac Surg Clin North Am.

[2] García Lorenzo J, Montoro Martínez V, Rigo Quera A, Codina Aroca A, López Vilas M, Quer Agustí M, León Vintró X (2017). Modifications in the treatment of advanced laryngeal cancer throughout the last 30 years. Eur Arch Otorhinolaryngol.

[3] Mulcahy CF, Mohamed ASR, Kanwar A, Hutcheson KA, Ghosh A, Vock D, Weber RS, Lai SY, Gunn GB, Group MLCW (2018). Age-adjusted comorbidity and survival in locally advanced laryngeal cancer. Head Neck.

[4] Zhou Y, Yang D, Yang Q, Lv X, Huang W, Zhou Z, Wang Y, Zhang Z, Yuan T, Ding X (2020). Single-cell RNA landscape of intratumoral heterogeneity and immunosuppressive microenvironment in advanced osteosarcoma. Nat Commun.

[5] Zhang G, Fan E, Yue G, Zhong Q, Shuai Y, Wu M, Feng G, Chen Q, Gou X (2019). Five genes as a novel signature for predicting the prognosis of patients with laryngeal cancer. J Cell Biochem.

[6] Cury SS, Lapa RML, de Mello JBH, Marchi FA, Domingues MAC, Pinto CAL, Carvalho RF, de Carvalho GB, Kowalski LP, Rogatto SR (2020). Increased DSG2 plasmatic levels identified by transcriptomic-based secretome analysis is a potential prognostic biomarker in laryngeal carcinoma. Oral Oncol.

[7] Nicolau-Neto P, de Souza-Santos PT, Severo Ramundo M, Valverde P, Martins I, Santos IC, Dias F, de Almeida ST, Ribeiro Pinto LF (2020). Transcriptome analysis identifies alcam overexpression as a prognosis biomarker in laryngeal squamous cell carcinoma. Cancers.

[8] Chang L-C, Lin H-M, Sibille E, Tseng GC (2013). Meta-analysis methods for combining multiple expression profiles: comparisons, statistical characterization and an application guideline. BMC Bioinformatics.

[9] Anderson WISD, Vesely KR (1989). Thyroid follicular carcinoma with pulmonary metastases in a beaver (Castor canadensis). J Wildl Dis.

[10] Wang P, Wang Y, Hang B, Zou X, Mao J-H (2016). A novel gene expression-based prognostic scoring system to predict survival in gastric cancer. Oncotarget.

[11] Goeman JJ (2010). L1 penalized estimation in the Cox proportional hazards model. Biom J.

[12] Tibshirani R (1997). The lasso method for variable selection in the Cox model. Stat Med.

[13] Wu G, Zhang M (2020). A novel risk score model based on eight genes and a nomogram for predicting overall survival of patients with osteosarcoma. BMC Cancer.

[14] Handisurya A, Rumpold T, Caucig-Lütgendorf C, Flechl B, Preusser M, Ilhan-Mutlu A, Dieckmann K, Widhalm G, Grisold A, Wöhrer A (2019). Are hypothyroidism and hypogonadism clinically relevant in patients with malignant gliomas? A longitudinal trial in patients with glioma. Radiother Oncol.

[15] Eng KHSE, Morrell K (2015). On representing the prognostic value of continuous gene expression biomarkers with the restricted mean survival curve. Oncotarget.

[16] Harrell FE, Lee KL, Mark DB (1996). Multivariable prognostic models: issues in developing models, evaluating assumptions and adequacy, and measuring and reducing errors. Stat Med.

[17] Mayr A, Schmid M (2014). Boosting the concordance index for survival data–a unified framework to derive and evaluate biomarker combinations. PLoS ONE.

[18] Xiang Y, Li C, Liao Y, Wu J (2019). An integrated mRNA-lncRNA signature for relapse prediction in laryngeal cancer. J Cell Biochem.

[19] Zhang G, Fan E, Zhong Q, Feng G, Shuai Y, Wu M, Chen Q, Gou X (2019). Identification and potential mechanisms of a 4-lncRNA signature that predicts prognosis in patients with laryngeal cancer. Hum Genomics.

[20] Fan L, Zhao R, Chen X, Liu Y, Tian L, Liu M (2022). Establishment of a non-squamous cell carcinoma of the larynx nomogram prognostic model and prognosis analysis. Auris Nasus Larynx.

